# Oral midazolam vs. intranasal dexmedetomidine plus oral midazolam for sedation of pediatric outpatients: a double-blinded randomized controlled trial

**DOI:** 10.1186/s12871-023-02289-5

**Published:** 2023-10-10

**Authors:** Juan Nie, Chanchan Chen, Jing Xie, Guicong Ding

**Affiliations:** 1 Shenzhen Children’s Hospital of China Medical University, Shenzhen, 518026 China; 2 Department of Pediatric Dentistry, Sichuan Hospital of Stomatology, Chengdu, 61000 China; 3 Department of stomatology, Shenzhen Children’s Hospital, China Medical University, Yitian Road 7019, Shenzhen, 518000 P.R. China

**Keywords:** Dental anxiety, Midazolam, Dexmedetomidine, Sedation, Pediatric dentistry

## Abstract

**Background:**

Moderate to deep sedation is required for dental treatment of children with dental anxiety. Midazolam is the most commonly used sedative, whereas intranasal dexmedetomidine is increasingly used in pediatric sedation.

**Objective:**

The aim of this trial was to compare the sedative efficacy of oral midazolam alone with that of intranasal dexmedetomidine plus oral midazolam during dental treatment of children with dental anxiety.

**Design:**

In total, 83 children (aged 3–12 years) scheduled to undergo dental sedation were randomized to receive oral midazolam (0.5 mg/kg) and intranasal placebo, or oral midazolam (0.5 mg/kg) plus intranasal dexmedetomidine (2 µg/kg). The primary outcome was the rate of successful sedation for dental treatment. Secondary outcomes were the onset time and adverse events during and after treatment. Data analyses involved descriptive statistics and nonparametric tests.

**Results:**

The rate of successful sedation was significantly higher in combination group (*P* = 0.007), although the sedation onset time was significantly longer in combination group (17.5 ± 2.4 min) than in monotherapy group (15.7 ± 1.8) (*P* = 0.003). No children required medical intervention or oxygen therapy for hemodynamic disturbances, and the incidences of adverse events had no significant difference between groups (*P* = 0.660).

**Conclusion:**

Combined treatment with oral midazolam (0.5 mg/kg) and intranasal dexmedetomidine (2 µg/kg) is more significantly effective for managing the behavior of non-cooperative children during dental treatment, compared to oral midazolam (0.5 mg/kg) alone. (Chinese Clinical Trial Registry: ChiCTR2100042300)

**Trial registration:**

ChiCTR2100042300, Clinical trial first registration date: 17/01/2021.

## Introduction

Children are a unique group of dental patients, such that invasive oral treatment of children is the most challenging clinical task for many dentists [1]. In many countries, the prevalence of dental anxiety in adolescents was estimated to range from 5–20% [2, 3]. Children’s anxiety concerning dental treatment may lead to behavioral management problems, which interfere with successful dental treatment [4]. To relieve children’s anxiety and manage their behavior, pharmacologic sedation and analgesia are commonly used.

Oral midazolam is an effective sedative agent for children undergoing dental treatment [4]. However, because of first-pass hepatic metabolism, it is difficult to accurately calculate the effective dose absorbed. Therefore, the sedative effect cannot be adjusted and titrated, causing large individual differences, and widely variable sedation success rate (e.g., from 30–70%) [4]. Accordingly, some children require additional sedatives. Previous reports have demonstrated that when buccal midazolam is added to intranasal dexmedetomidine, the successful sedation rate for computerized tomography (CT) and/or auditory brainstem response test (ABR) is higher than oral chloral hydrate or intranasal dexmedetomidine in children [5, 6]. However, this combination has only been studied in children undergoing short and non-stimulating procedures. To the best of our knowledge, there is a lack of well-designed trials concerning the efficacy of intranasal dexmedetomidine combined with oral midazolam in uncooperative children undergoing dental treatment. This trial evaluated the efficacy of combined treatment with dexmedetomidine and midazolam, compared to oral midazolam alone.

## Materials and methods

This study protocol was approved by the Ethics Committee of Shenzhen Children’s Hospital of China Medical University (IRB no. 201,904,802). The rights of the participants were protected, and at least one of each patient’s parents or a legal guardian signed a statement of informed consent before the patient was enrolled. All participating children were verbally informed of the relevant trial protocol in advance. The study was performed in accordance with the World Medical Association Declaration of Helsinki ethical principles for medical research involving children. The participant recruitment began on January 20, 2021, and the study was completed on May 31, 2021.

### Participants and study setting

Children were enrolled in the trial only if they were younger than 12 years of age, within the normal weight range, had American Society of Anesthesiologists physical status I or II, and required dental treatment in > 1 teeth. Exclusion criteria were a known medical history of neurological or cognitive changes, allergy to the study drugs, arrhythmia, heart disease or organ dysfunction, and/or any treatment with sedatives; history of respiratory infection or obstructive disease in the past week; and withdrawal/refusal to participate. The participants were recruited for dental treatment under sedation because of their uncooperative behavior confirmed by two dentists after two previous dental examinations. If a child was willing to undergo a dental examination, s/he will be scheduled for treatment without sedation and excluded from the study. All patients fasted 6 h for solids and 2 h for clear liquids.

### Randomization and blinding

For random grouping, a computerized random number generator was used. When parents or guardians provided written informed consent for their children to participate in this study, the computer software randomly generated a code (pre-defined code group) and participants were divided into two groups using these codes. The randomization codes were kept in sealed envelopes in order and held by the anesthesiologist. On the day of treatment, the anesthesiologist and nurse opened the envelope and prepared the study drugs according to the allocated group.

Only the anesthesiologist and nurse involved in sedative administration had information concerning the sedative administered to each participant. If any adverse event occurred, clinical intervention was immediately implemented. The participants, their parents/guardians, the pediatric dentist and nurse involved in dental treatment, observer, data collector, and statistical analyst were unaware of each participant’s group assignment.

### Interventions

The participants were randomly assigned to two groups the dexmedetomidine- midazolam (DM) group received 2 µg/kg intranasal dexmedetomidine (maximum 100 mcg) combined with 0.5 mg/kg oral midazolam (maximum 20.0 mg). The midazolam (M) group received 0.5 mg/kg oral midazolam (maximum 20.0 mg) combined with intranasal normal saline. The dexmedetomidine used in this trial was free of preservatives, with a concentration of 0.1 mg/mL: (You Bi Tuo; Yangzi River Pharmaceutical, Jiangsu, CHN, China). Undiluted dexmedetomidine was drawn into a 1 mL tuberculin syringe and administered using a nebulizer (MAD300, Wolf Tory Medical, Salt Lake City, ST, United States). For oral midazolam, an intravenous formulation (5 mg/mL; Nhwa Pharma Corporation, Jiangsu, CHN, China) was mixed with fruit juice using a volumetric ratio of 1:2.

Before dental sedation, an anesthesiologist assessed each participant’s general condition, confirmed whether they met the inclusion criteria, and administered the sedation in accordance with the participant’s weight and randomization status. The anesthesiologist initially sprayed either the appropriate dexmedetomidine dose (for DM group), or an equal volume of normal saline (for M group), into both nostrils of each participant at a uniform rate. To maximize intranasal drug absorption, participants were encouraged to maintain the supine position for 1–2 min. After 10 min the anesthesiologist administered oral midazolam fruit drink for participants in the M and DM groups at a predetermined dose.

Ramsay scale score[RSS]^(7)^ was evaluated every 2 min after administration. After sedative onset (Ramsay scale score [RSS]^(7)^ of 2–3 points) (Table 1), a nurse escorted each participant to the designated dental clinic. To avoid accidental fall, s/he was arranged to lie on dental chair with a seat belt (a minimal degree of physical restraint). In order to avoid the fear of separation from their parents, parents were allowed to stay with them during treatment. The same pediatric dentist and nurse completed the corresponding dental treatment (e.g., filling, root canal treatment, or crown restoration) under local infiltration anesthesia and rubber dam isolation. Before the child fell asleep, in accordance with each participant’s specific situation, the pediatric dentist used appropriate behavior management techniques (e.g., distraction, positive reinforcement, or nonverbal communication). The duration of treatment was consistently < 45 min (1 to 3 teeth were treated at a time). Each participant’s blood pressure, heart rate and oxygen saturation were continuously monitored throughout the treatment process. Supplemental oxygen was given if SpO_2_ was below 94%. If the dental treatment couldn^’^t be completed due to patient’s strenuous movement, another sedation or general anaesthesia was arranged on another day. After treatment, each participant was observed in the sedation recovery room for > 30 min until they met the discharge criteria [7]. One trained independent research observer was involved in observation and data collection.

**Table 1:** Evaluation scales

**Ramsay sedation score**	
1	patient anxious and/or agitated
2	patient co-operative, orientated, and quiet
3	patient only responds to orders
4	a brisk response to a light glabellar tap or loud auditory stimulus
5	a sluggish response
6	no response
**Frankl scale**	
1	complete rejection of treatment
2	relative rejection
3	ability to cooperation
4	very cooperation.
**Houpt scale**	
1	aborted: no complete treatment
2	poor: treatment interrupted, only part of the treatment completed
3	fair: treatment interrupted, but eventually all completed
4	good: difficult, but all treatment performed
5	very good: some limited crying or movement
6	excellent: no crying or movement

### Outcomes

The primary outcome was the rate of successful sedation for dental treatment. Secondary outcomes were the dental treatment success rate, intraoperative or postoperative adverse events and parental satisfaction with the sedation treatment.

The RSS was used to evaluate each participant’s sedation status during dental treatment [8]. Patient behavior during sedation was evaluated using the Frankl scale [9] (Table 1). Evaluation of completion of treatment under sedation was evaluated by the Houpt scale [10] (Table 1). The above scales were evaluated every 5 min after treatment. The score with the most occurrences was recorded as the final score of the scale. Frankl scale scores of 1 or 2 were considered sedation failure, and scores of 3 or 4 were considered sedation success. Furthermore, Houpt scale scores of 1 or 2 were considered treatment failure, and scores of 3–6 were considered treatment success. These evaluations were performed by the blinded observer previously trained by theoretically and practically.

Unexpected and undesirable responses to sedatives that threaten or cause patient injury or discomfort were defined as adverse events, in accordance with the World Society of Intravenous Anesthesia International Sedation Task Force Tool [11, 12]. Such events included agitation, drowsiness, motor imbalance, dizziness, respiratory distress, nausea, vomiting, gastrointestinal symptoms, and any other unexpected or undesirable responses to sedatives that could threaten or cause patient injury or discomfort [11]. The blinded observer registered all adverse events during and after the dental sedation procedure (both in the procedure room and recovery room). 24 h after treatment, the blinded observer interviewed the participant’s parents by anonymous e-mail questionnaires about whether their child had any adverse events, whether the parents were satisfied with the sedation treatment (very satisfied, generally satisfied or dissatisfied). The above procedures did not change after the start of the trial.

### Statistical analyses

In our pilot study, the success rate of oral midazolam was approximately 50%, and that of intranasal dexmedetomidine combined with oral midazolam was approximately 80%. Based on the success rate in pilot study, the sample required was at least 38 participants per group, with a power of 80% at a 5% level of type 1 error.

EpiData (version 3.1; EpiData Association, Odense, Denmark) was used for data entry and the statistical software SPSS Statistics (version 26.0; IBM Corp., Armonk, NY, USA) was used for data analyses. The significance threshold was set at 5%. Continuous data with normal distributions were expressed as means ± standard deviations, and the t-test was used for comparisons between groups. Continuous data without normal distributions were expressed as medians (interquartile ranges), and the Mann–Whitney U test was used for comparisons between groups. Categorical data are expressed as numbers (percentages), and the chi-square test (Pearson’s or Fisher’s exact test) was used for comparisons between groups.

## Results

**Fig. 1:**
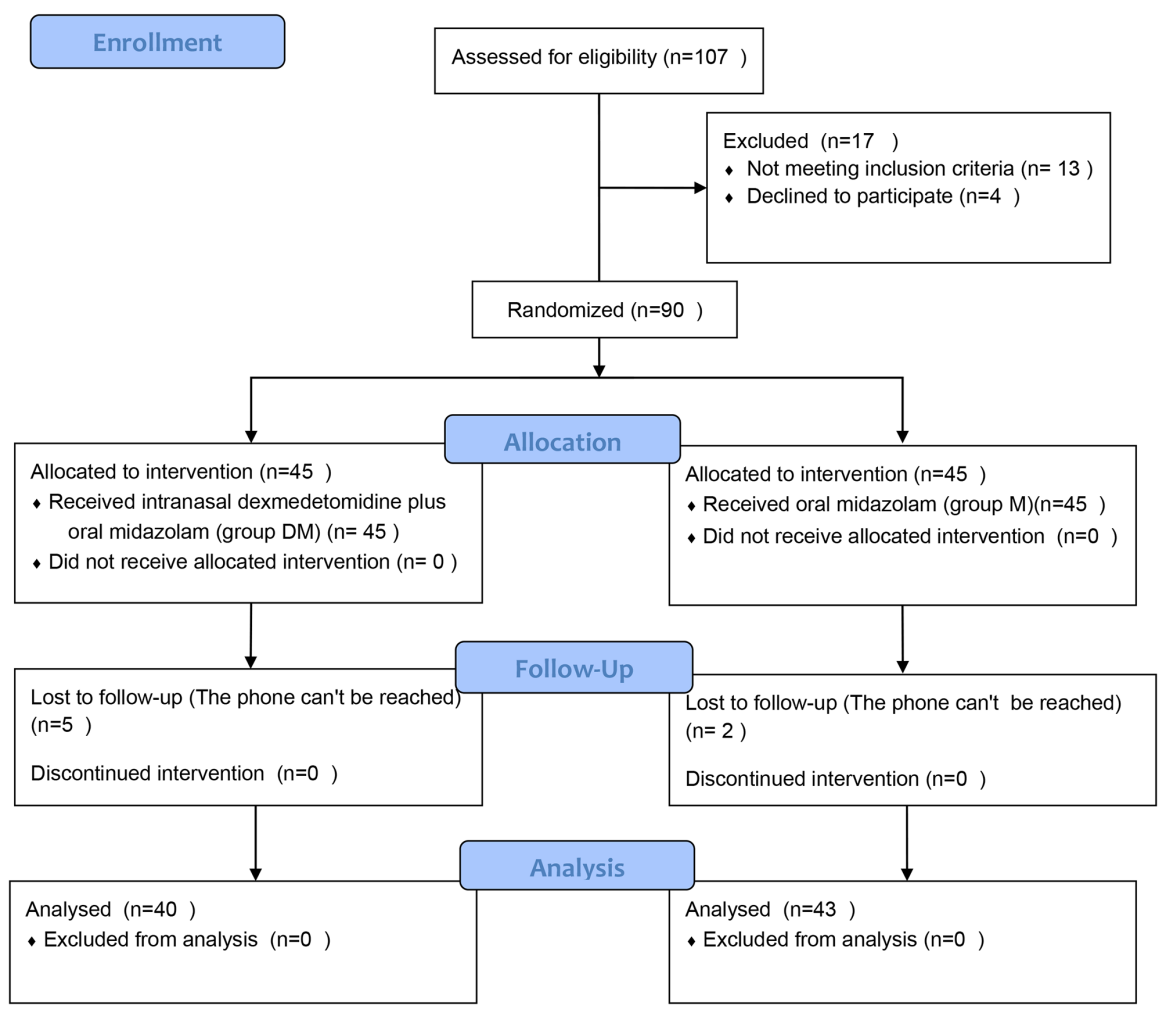
CONSORT 2010 Flow Diagram

In total, 107 dental patients aged 3 to 12 years were recruited from January 20, 2021 to February 20, 2021. Among them, 13 patients did not meet the inclusion criteria and were excluded, and four patients refused to participate. Accordingly, 90 patients were randomly divided into two groups. However, two participants in M group and three participants in DM group requested to withdraw during the study, and two participants in DM group were lost to follow-up. Therefore, 83 participants completed the final analyses, as illustrated in Fig. 1.

The average age of the 83 participants (32 boys; 38.6%) was 6.1 years (standard deviation, 2.7 years). The characteristics of the participants in the baseline and clinical characteristics are presented in Table 2. The total time of treatment was similar (*P* = 0.318), but the onset time of sedation was significantly longer in DM group (17.5 ± 2.4 min) than in M group (15.7 ± 1.8) (*P* = 0.003, Table 2).

**Table 2:** Comparison of demographic information and clinical characteristics of recruited participants for the two groups

Variables	M group	DM group	P
Number of Children	43	40	
Age(year)	5.7 ± 2.3	6.4 ± 2.9	
Sex(M/F)	28/15	23/17	
Wight (Kg)	20.4 ± 6.7	23.0 ± 9.5	
ASA classification (I/II)	43/0	37/3	
Use local infiltration anesthesia (yes/no)	24/19	21/19	1
Onset for sedation (min)	15.7 ± 1.8	17.5 ± 2.4	0.003
Total treatment time (min)	35.6 ± 7.8	38.8 ± 9.5	0.318

values in number mean ± standard deviation (SD)

**Fig. 2:**
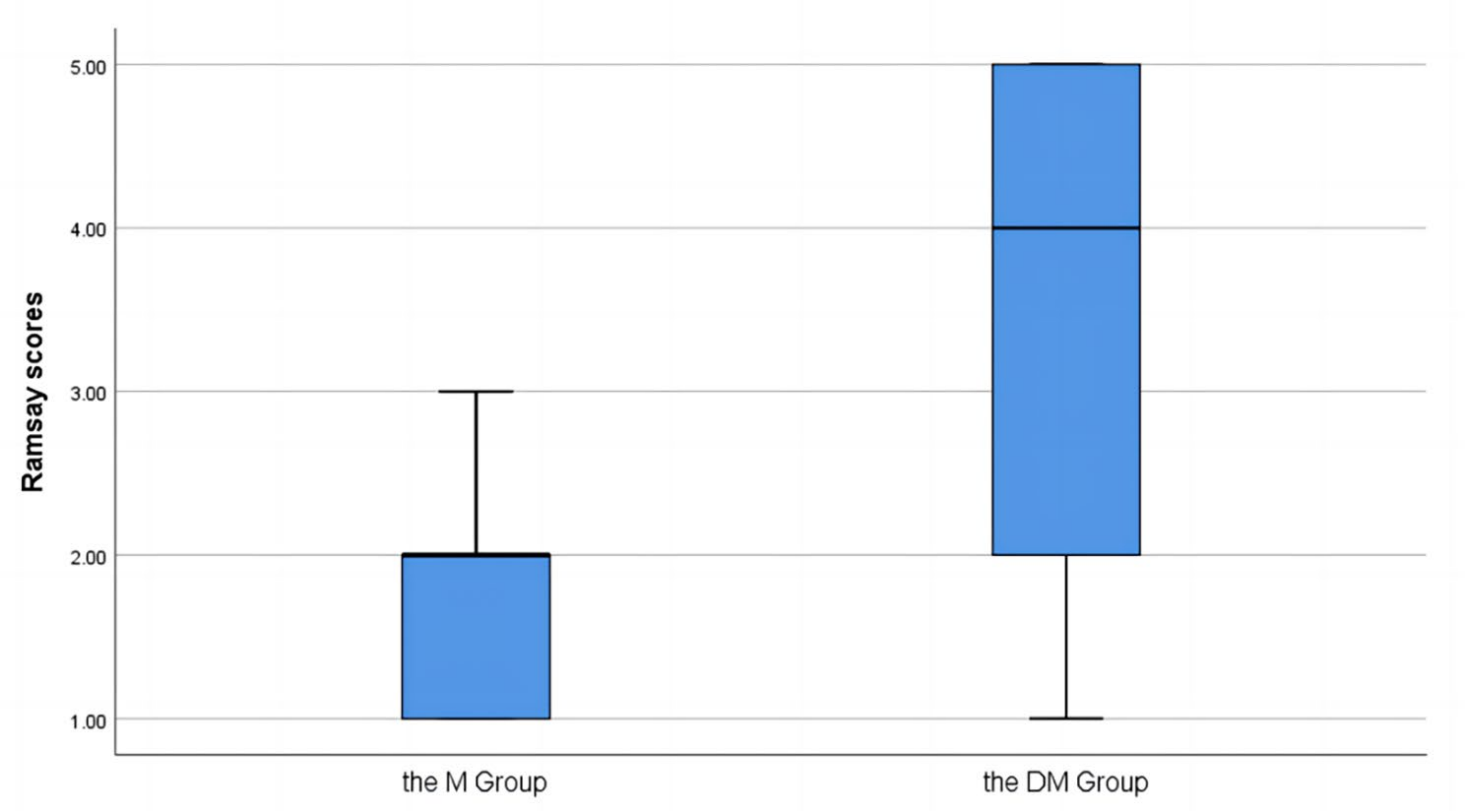
Ramsay scores for two groups

**Fig. 3:**
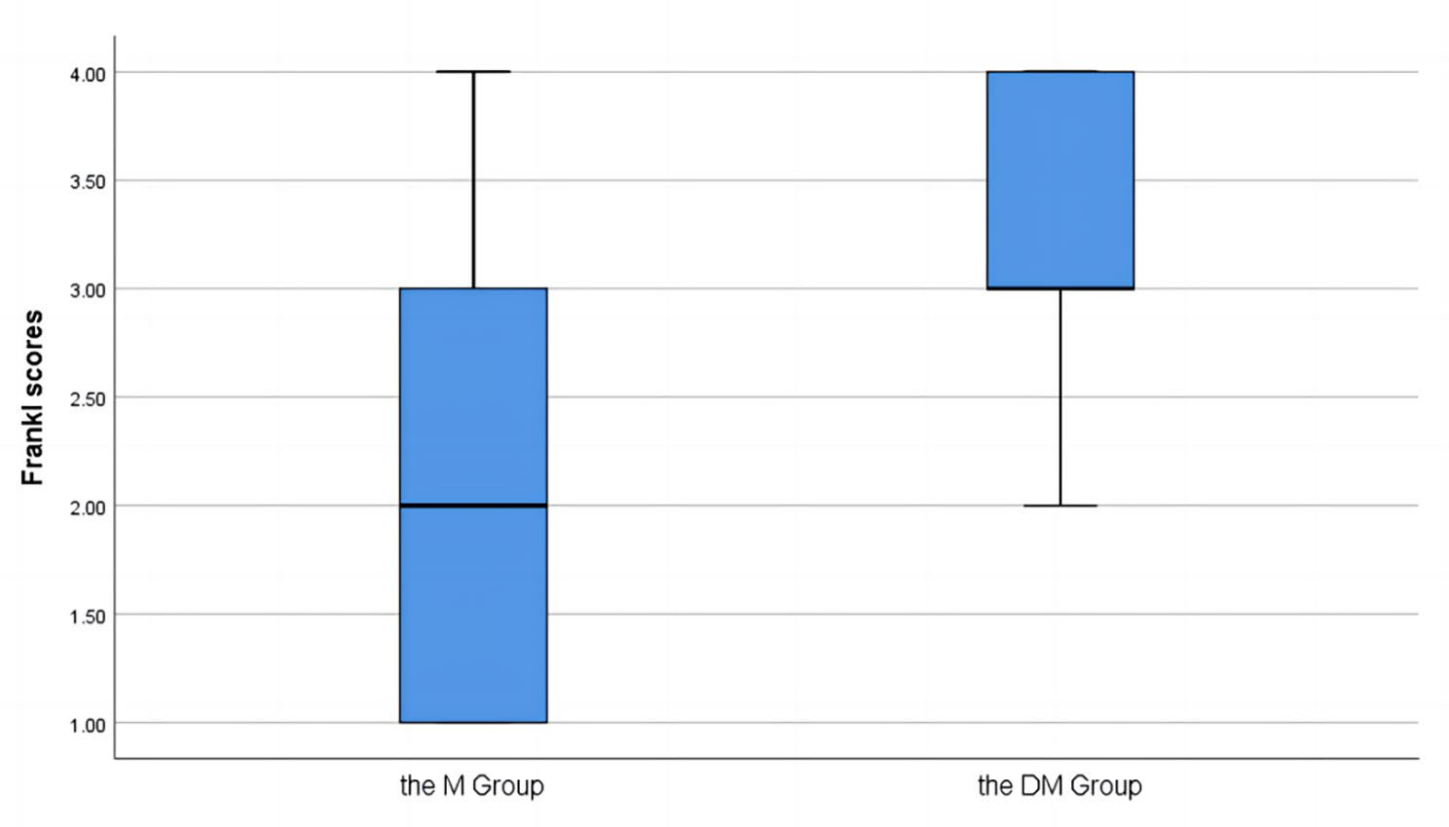
Frankl scores for two groups

**Fig. 4:**
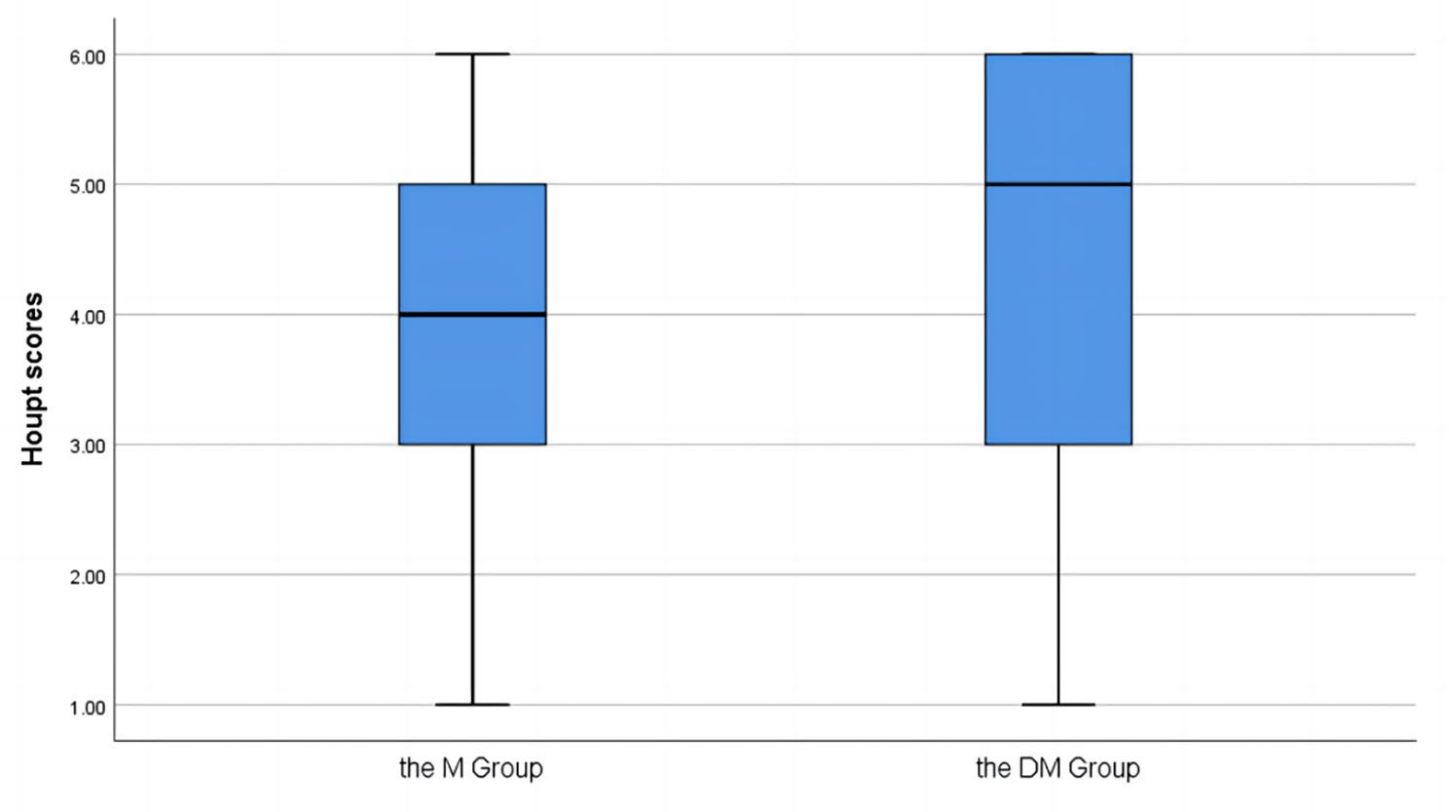
Houpt scores for two groups

The Mann–Whitney U test was used to examine potential differences between groups in the RSS, Frankl scale, and Houpt scale (Figs. 2, 3 and 4). Medians of the RSS values for DM group [4 [2, 5]] and M group [2 [1, 2]] were significantly different (*P* < 0.05), indicating that the participants had better sedative effects in DM group than in M group. The Frankl scale scores for DM group [3 [3, 4]] and M group [2 [1, 3]] were significantly different (*P* = 0.008), indicating that the participants had better treatment compliance in DM group than in M group. The Houpt scale scores for DM group [5 [3, 6]] and M group [4 [3, 5]] were significantly different (*P* = 0.033), indicating that the participants experienced more efficient treatment in DM group than in M group.

Among the 43 participants in M group, 21 (48.8%) were successfully sedated with scheduled dental treatment, but 22 (51.2%) were not. Among the 40 participants in DM group, 31 (77.5%) received successful sedation with the corresponding dental treatment, but nine (22.5%) were not successfully sedated. The odd ratio (95% CI) for successful sedation between oral midazolam plus intranasal dexmedetomidine and oral midazolam estimated to be 3.61 (1.39-9.36), *X*^*2*^ = 2.895, *P* = 0.007. In M group, 40 participants (93.0%) completed the corresponding dental treatment, but three participants (7.0%) only partially completed the treatment. In DM group, 45 participants (100%) completed the scheduled dental treatment. But the success rate of treatment was not different between the two groups (*X*^*2*^ = 2.895, *P* = 0.242).

During treatment, the blood oxygen saturation of the two groups fluctuated within the normal range, but no oxygen saturation decreased below 95%. In DM group, one participant had bradycardia (heart rate dropped to 60 beats per minute in the dental procedure), another participant occurred hypotension (systolic blood pressure dropped to 90 mmHg in procedure). However, no patient required the intervention of the anesthesiologist, both patients returned to normal within a minute. Five children (12.5%) in DM group, showed signs of lethargy, and none of this sign was seen in M group (*P* = 0.023, Table 3). The overall incidence of adverse events was 16.3% in M group, and 20.0% in DM group. However, there were no significant differences in the incidence of adverse events between the two groups (*P* = 0.660). None of the 19 participants with adverse events received specialized intervention. All adverse event symptoms were relieved after resting.

**Table 3:** absolute and relative frequencies of adverse events related to sedative groups

Variables	M group	DM group	*P*
delirium	2(4.7%)	0	0.495
diplopia	1(2.3%)	0	1.00
nausea and vomiting	2(4.7%)	0	0.495
agitation	2(4.7%)	1 (2.5)	1.00
loss of coordination	0	1(2.5%)	0.482
bradycardia	0	1(2.5%)	0.482
lethargy	0	5(12.5%)*	0.023

*Significant differences (P < 0.05)

Upon questionnaires interviewed at 24 h after discharge, the parents reported that all adverse events had disappeared without any new adverse events. In M group, the parents of 23 participants (53.5%) were very satisfied with the sedation treatment, the parents of 20 participants (46.5%) were generally satisfied, and no parents were dissatisfied (Table 4). In DM group, the parents of 34 participants (85%) were very satisfied, the parents of four participants (10%) were generally satisfied, and the parents of two participants (5%) were dissatisfied. The parental satisfaction of DM group was significantly higher than that of M group (*P* = 0.001).

**Table 4:** Parents’ attitudes towards sedation treatment

Variables	M group	DM group	*P*
very satisfied	23(53.5%)	34(85.0%)	0.001*
generally satisfied	20(46.5%)	4(10.0%)	
dissatisfied	0	2(5.0%)	

*Significant differences (P < 0.05)

## Discussion

To identify the safer and more effective sedative for use in pediatric dentistry, various dosages and routes of administration have been explored for multiple sedative drugs [13–16]. Among them, dexmedetomidine is increasingly used in pediatric sedation. Dexmedetomidine can be administered by intravenous, oral, mucosal, and intramuscular routes [17]. In this study, we used nasal spray administration. In contrast to oral administration, nasal spray administration avoids first-pass hepatic metabolism, providing more reliable absorption [18]. In contrast to intravenous and intramuscular injection, intranasal dexmedetomidine is a noninvasive method of administration, which does not stimulate the nasal mucosa, avoids eliciting patient fear, and is more acceptable to children. Although bradycardia and hypotension are common side effects of dexmedetomidine sedation, intervention is rarely required [5], as observed in this study. In our clinical experience, we found that sudden arousal in response to stimulation, such as pain or sound stimulation in the procedures, may be a disadvantage of dexmedetomidine as a sole sedative, which resulted in a clinically significant rate of sedation failure, corresponding with previous studies [6].

Dexmedetomidine can be used as an adjunct to benzodiazepines [19]. Some prospective clinical trials have shown that dexmedetomidine combined with midazolam is a safe and effective approach, with deeper level of sedation and greater success rate of sedation, without increasing the incidence of adverse events, in non-painful examinations (e.g., CT or ABR test) [20, 21]. In Li et al. [5] study, intranasal dexmedetomidine at 3 µg/kg plus buccal midazolam at 0.1 mg/kg was associated with higher sedation success rate with deeper level of sedation attained when it was compared with oral chloral hydrate at 50 mg/kg for auditory brainstem response testing in healthy children aged between 2 months and 6 years. Recently, Li et al. [6] found that the sedation success rate was 65.4% (89 out of 136) for non-painful procedural sedation in children with autism who had intranasal dexmedetomidine (3 µg/kg) and 83.5% (116 out of 139) who had intranasal dexmedetomidine (3 µg/kg) plus buccal midazolam (0.2 mg/kg). However, the combination regimen is rarely applied in dental treatment and other invasive operations. The results of this study showed that the RSS, Frankl scale, and Houpt scale scores were significantly greater in DM group than M group. Furthermore, the level of sedation was deeper in DM group, and the patient compliance was better, such that the overall treatment process was more efficient without any additional respiratory side effects. The treatment completion rate in both groups was > 90% (under the sedation and physical protective restraint), and the sedation success rate in DM group was significantly greater than that in M group. In addition, parents were more satisfied with this combined medication plan. All above results showed that the combination of dexmedetomidine and midazolam was a safe and effective alternative sedation. This combination may be a useful sedation alternative regimen in children undergoing longer duration and higher level of stimulation.

Our results confirmed that the onset time of sedation was significantly longer in DM group (17.5 ± 2.4 min) than in M group (15.7 ± 1.8) (*P* = 0.003, Table 2). This is a statistically significant difference of about two minutes, but the clinically significant difference of two minutes seems not to be significant. The incidence of lethargy was higher in the DM group. But there were no significant differences in the incidence of adverse events between the two groups, no specialized clinical intervention was administered, and no serious adverse events occurred. However, the dose and interval of the combination are not necessarily the optimal schedule. This study may be a preliminary exploration. We will further explore the optimal drug dose for the combination and it’s clinically relevant will be explored in the future.

The strengths of this study included its enrollment of patients who received sedation for the first time, to avoid the influence of past negative dental sedation experiences on the results of the trial. Notably, an unpleasant sedation intervention may affect the success of subsequent interventions [22], thereby limiting comparisons with parallel, randomized clinical trials. Furthermore, this prospective, double-blind randomized clinical trial used the RSS, Frankl scale, and Houpt scale for the evaluation of sedation, thereby comprehensively evaluating the effects of sedation from three perspectives: sedation depth, patient compliance, and treatment completion, avoiding interference from subjective factors. The results will be helpful to guide doctors to select the appropriate administration route of sedation. Based on the results of this study and our clinical experience, it is suggested that the combination of dexmedetomidine and midazolam may be more favorable for children with severe dental anxiety, needing deep sedation for treatment. When the sedative effect of oral midazolam alone is not well, combining intranasal dexmedetomidine could be considered. Given the difference in onset times for midazolam and dexmedetomidine and the overall long onset time, good timing and planning is required in order to get timely through the operation schedule. This might not be applicable to all institutions especially if staff is limited or turnover of patient is highly.

However, this study was limited in that we relied on the provision of post-sedation data from parents. This may have permitted bias and variation in feedback, due to differences in parental understanding of the observer’s questions. In addition, this study had a small patient sample and we did not stratify children according to psychological development. However, psychological characteristics may affect the sedation behavior of children [23]. Therefore, future studies should use larger sample sizes and involve multiple institutions in randomized assessments that involve age stratification.

## Conclusion

Oral midazolam at 0.5 mg/kg plus intranasal dexmedetomidine at 2 µg/kg was associated with higher sedation success rate for managing the behavior of non-cooperative children during dental treatment, compared to oral midazolam (0.5 mg/kg) alone. This combination of oral midazolam and intranasal dexmedetomidine may be a safe and useful alternative sedative regimen in children undergoing long duration and high level of stimulation.

## Data Availability

The datasets generated during and/or analysed during the current study are available by contacting the first author via e-mail 13,518,289,033@163.com.
